# Collective Knowledge Used to Unveil Cardiovascular Injury Emerged during COVID-19

**DOI:** 10.3390/ijms23095178

**Published:** 2022-05-06

**Authors:** Atsushi Tanaka, Koichi Node

**Affiliations:** Department of Cardiovascular Medicine, Saga University, Saga 849-8501, Japan; node@cc.saga-u.ac.jp

Two years have passed since the unprecedented breakout of the global pandemic of the coronavirus disease COVID-19, which began at the end of 2019. So far, we have learned much about COVID-19 and have tried to overcome this pandemic. It is currently true that the situation surrounding COVID-19 has rapidly and greatly changed with the development of vaccines and therapeutics. However, it is also true that clinicians and scientists were greatly challenged by this unknown disease.

In an initial report from Wuhan, the prevalence of old age and comorbidities, such as hypertension, diabetes, and coronary heart disease was more frequently observed in non-survivors than in survivors of COVID-19, suggesting that people with some conventional risk factors of cardiovascular disease are at a high risk of fatal outcomes where COVID-19 is concerned [1]. Additionally, most frontline researchers and clinicians fighting against COVID-19 reported that some deaths of individuals infected with severe acute respiratory syndrome coronavirus 2 (SARS-CoV-2) might be partially explained by non-respiratory fatal conditions such as microcirculatory insufficiency in multiple organs and cardiovascular-related complications [2,3]. These observations made us recognize the unusual clinical characteristics of COVID-19 compared with the common respiratory viral infections, such as influenza.

In May 2020, the first report indicating that SARS-CoV-2 directly infected systemic vascular endothelial cells in the kidney, heart, intestine, and lungs was published [4]. This endothelial cell infection caused diffuse endotheliitis in several organs accompanied by excessive inflammatory and immune responses. The report showed that endothelial dysfunction, acutely evoked by SARS-CoV-2 infection, could secondarily impair systemic microcirculatory function and balance of the coagulation–fibrinolysis system, leading to systemic hypercoagulability, rapidly progressive multiple organ failure, and death. However, little was initially understood about the detailed mechanisms of cardiovascular injuries that emerged during the COVID-19 pandemic. Therefore, in May 2020, we launched a Special Issue, “Cardiovascular Injuries in Severe Respiratory Infectious Diseases”, to highlight and collect scientific papers to extensively explore the molecular mechanisms by which the COVID-19 and SARS-CoV-2 damage the cardiovascular system and cause cardiovascular complications. We, along with the help of excellent authors and reviewers, have published nine papers in the past two years. In this editorial, we would like to express our gratitude to them and introduce them very briefly; please refer to each publication for details.

Firstly, Joshi et al. [5] examined the possible molecular mechanisms underlying the augmentation of COVID-19 pathologies due to obesity associated metabolic disturbances. They focused on the biological molecules associated with palmitic acid and COVID-19, and the 35 molecules affected by both were obtained from ingenuity pathway analysis, i.e., converging on the effects of several pathways. Their study also found that one of the top 10 canonical pathways affected by palmitic acid was the coronavirus pathogenesis pathway which is mediated by several inflammatory mediators. In addition, they found that several molecules overlapping palmitic acid and COVID-19 were associated with an increase in the activity of the angiotensin-converting enzyme 2.

Secondly, Venter et al. [6] compared the plasma levels of several circulating biomarkers, including serum ferritin and P-selectin, among COVID-19 patients and healthy volunteers. They found that a decrease in soluble P-selectin levels might be an important early marker of severe vascular disease risk in COVID-19. In addition, they investigated the structure of platelets and erythrocytes using scanning electron microscopy. They found an association between increased soluble P-selectin and serum ferritin levels with pathological changes in both platelets and erythrocytes. These changes can play pathological roles in the development of thrombotic microangiopathy complicated by COVID-19.

Thirdly, Karmouty-Quintana et al. [7] reviewed the unique pathophysiology of COVID-19-induced pulmonary vasoconstriction and acute respiratory distress syndrome (ARDS), owing to the unique molecular features of vascular injuries and cytokine release storm. Following SARS-CoV-2 infection, hypoxia can promote sustained pulmonary vasoconstriction and pulmonary edema, resulting in a vicious cycle. In addition, increased inflammatory cytokine release during a cytokine storm also promoted pulmonary vasoconstriction and vascular remodeling. These pathologies are the hallmarks of pulmonary hypertension. Therefore, they concluded that pulmonary hypertension in COVID-19-induced ARDS represents an important target for disease amelioration.

Fourth, Yamaoka-Tojo [8] first proposed the novel concept of systemic inflammatory-reactive microvascular endotheliopathy (SIRME), which plays a key pathophysiological role in COVID-19. SIRME is caused by conventional cardiovascular risk factors and COVID-19-induced damage to the vascular endothelial glycocalyx that covers the surface of vascular endothelial cells. It is characterized by strong inflammation, vascular endothelial damage, and multiple organ failure. In COVID-19, it is necessary to identify clinical biomarkers that can reflect the severity of SIRME and potential treatments to protect vascular endothelial cells and prevent the progression of SIRME.

Fifth, Lee et al. [9] investigated the behavior of inflammation-related genes in the human-induced pluripotent stem cell-derived cardiomyocytes infected with SARS-CoV-2. They found that SARS-CoV-2 infection upregulates the expression of proinflammatory cytokines, including tumor necrosis factor-α (TNF-α), followed by enhancement of viral entry-related proteins, such as ACEs, and that the neutralization of TNF-α ameliorated this response. Their findings suggest that TNF-α plays a pivotal role in triggering the cytokine storm and myocardial injury in COVID-19.

Sixth, Homme et al. [10] investigated the pathological roles of matrix metalloproteinase-9 (MMP-9) and blood–heart barrier (BHB) leakage in heart failure (HF) and viral myocarditis using a mouse model. They found that activated MMP-9 causes endocardial endothelium dysfunction resulting in BHB leakage in the HF mouse model. Interestingly, hydroxychloroquine treatment alleviated MMP-9 activation, BHB leakage, and cardiac phenotypes of HF, suggesting that this pathological process could be a potential therapeutic target, even in COVID-19-induced cardiac injury.

Seventh, Nakano et al. [11] reviewed the current understanding of the mechanisms involved in the development of cardiovascular complications and fatal outcomes in the acute phase of COVID-19. They speculated that SARS-CoV-2-induced endothelial dysfunction caused an increase in platelet aggregation and hypercoagulability, resulting in thromboembolic events. Additionally, increased arterial stiffness in the vascular medial layer can also lead to heart failure, arrhythmia, and multi-organ dysfunction.

Eighth, Maruhashi and Higashi [12] summarized the putative mechanisms of endothelial dysfunction in patients with COVID-19. A combination of direct SARS-CoV-2 infection of endothelial cells and a cytokine storm originating in the lungs synergistically causes endothelial cell injury, resulting in endothelial dysfunction and subsequent cardiovascular complications. Thus, the mechanism of cardiovascular complications originating from COVID-19-induced vascular endothelial dysfunction was elucidated.

Finally, Higashikuni et al. [13] reviewed the cutting edge insights of dysregulated immune responses, platelet hyperactivation, and endothelial dysfunction in the cardiovascular pathogenesis of COVID-19. In particular, the authors emphasized the diverse mechanisms of cardiovascular injury in COVID-19 and the need to urgently establish treatment strategies for the multifaceted and complex pathophysiology of the disease.

Unfortunately, the full extent of the pandemic is not yet understood. This Special Issue ends, but the fight against the pandemic continues. However, the fact that so much knowledge has been gained over such a short period is a major milestone in the ongoing fight against this pandemic and any unknown pandemics that may arise in the future. We have used collective knowledge to unveil cardiovascular injury in emerging infectious diseases, which was not fully possible 100 years ago. We believe that our efforts can help overcome this pandemic. Once again, we are grateful to the contributors for their interest and hard work in this Special Issue.
